# Families’ perspectives and the development of an educational program for diabetes in schools

**DOI:** 10.1016/j.jped.2026.101512

**Published:** 2026-02-28

**Authors:** Laura Cudizio, Isadora R. Brambilla, Kayleigh G. Marques de Araújo, Raquel G. Lot, Tayana O. Martins, Raquel R. Biasi, Giuliane Galeote, Aline D. Costa-Riquetto, João Eduardo N. Salles, Luis Eduardo Calliari

**Affiliations:** Faculdade de Ciências Médicas da Santa Casa de São Paulo, Departamento de Pediatria, Divisão de Endocrinologia Pediátrica, São Paulo, SP, Brazil

**Keywords:** Diabetes mellitus, type 1, Child, Adolescent, Schools, Health education, Brazil

## Abstract

**Objective:**

The number of children with type 1 diabetes (CwD) is rising globally, increasing demand for school-based support. The primary objective was to assess parents’ perceptions of the current state of diabetes care in schools for CwD. The secondary objective was to gather feedback on a comprehensive educational program offered to participants.

**Methods:**

Families of CwD attending an outpatient clinic completed a survey about diabetes care offered by schools. A comprehensive diabetes educational program was developed, comprising a mobile application, website, and printed diabetes management plan. The program was presented to the families to facilitate communication with school staff.

**Results:**

A total of 107 families participated (mean age of students 10.7 ± 3.9 years; mean age at diagnosis 5.4 ± ± 3.3 years). Most were from the São Paulo metropolitan area (94.3%; 27 cities) and attended public schools (89.7%). Mean HbA1c was 8.8% ± 2.1 (75). Family members often provided in-school insulin support (25.2% overall; 55% of children < 6 years; *p* < 0.05). Nearly half of caregivers (45.8%) reduced working hours due to diabetes care (55.2% for children < 6 years; 0.05). Most families (73.8%) reported schools had little or no knowledge of diabetes, and 31.8% were dissatisfied with the care received. The possibility of having diabetes material specifically directed to schools was valued by the families and patients.

**Conclusion:**

Families of CwD in the São Paulo metropolitan area face challenges in school settings. Providing educational materials in different formats is necessary, but strategies are needed to involve school staff in the management actively.

## Introduction

The International Diabetes Federation (IDF) Atlas estimated that in 2024, 9.15 million people worldwide had type 1 diabetes (T1D), including 1.0 million under 15 years [1]. In 2025, Brazil ranked fourth globally for T1D in those under 20, with 101,000 cases and an incidence of 22.3 per 100,000 under 15 [2].

The number of children and adolescents with T1D (CwD) in schools is also expected to increase, highlighting the need for diabetes care support as they spend much of their day there. The minimum expected for T1D care at school is the right to attend safely and receive appropriate medical treatment during all school activities. Key stakeholders in managing a student’s diabetes at school include guardians, school personnel, and healthcare providers [3,4].

There is a clear gap in the literature on school staff preparedness to support CwD. Available studies consistently show that teachers have insufficient knowledge, and parents express concern about the lack of information and a lack of confidence in managing diabetes at school [5,6]. In addition, few reliable, evidence-based materials exist for schools, limiting their ability to provide safe and inclusive care. These gaps highlight the need for structured interventions that combine accessible resources with tailored training to empower school communities.

According to *Instituto Nacional de Estudos e Pesquisas Educacionais Anísio Teixeira* (INEP), São Paulo had 7,856 schools in 2023. Previous attempts to assess school personnel’s knowledge of diabetes or to provide in-person training reached relatively few staff, many of whom had never had a student with diabetes [[6], [7], [8]]. To address the challenge of training such an extensive school system, the authors developed a comprehensive educational program on diabetes care in schools, targeting school personnel, which included a printed diabetes management plan (DMP), a website, a mobile app (APP), and on-site training.

The primary objective was to assess parents’ perceptions of the current state of diabetes care in schools for CwD. The secondary objective was to gather multi-stakeholder feedback on a comprehensive educational program.

## Methods

### Study population

All families of CwD patients from the Pediatric Diabetes Outpatient Clinic at Santa Casa de São Paulo who met the eligibility criteria were invited to participate during routine visits. Eligible participants were caregivers of children with diabetes attending school (daycare, preschool, elementary school, middle school and high school) in 2023. All enrolled participants were invited to complete the follow-up survey.

All professionals from participating children’s schools were eligible to attend the on-site training sessions and were provided with links to access digital platforms.

### Study design

Caregivers completed a questionnaire, administered by a healthcare professional, to assess their perceptions of school-based diabetes care. Clinical and laboratory data were extracted from medical records, and an individualized Diabetes Management Plan was developed for each child.

After the initial assessment, families received an educational package on school diabetes care and were instructed to deliver it to the child’s school. A follow-up questionnaire four months later evaluated perceived changes in school diabetes care.

### Educational package and training

The educational package included a hard copy of a DMP, website instructions, a QR code to download the APP, information on scheduling on-site training, and researcher contact details.

The IDF-KiDS and Diabetes Project´s material served as the basis for developing the educational material [9]. On digital platforms, users could select an area of interest to deepen their knowledge, such as diabetes diagnosis, adapting the school for a CwD, glycemic control, insulin administration, diet, hypoglycemia, hyperglycemia, physical activity, extracurricular activities, and managing diabetes at school. Each session ends with four questions to reinforce learning. The content was available for free online at diabeteseaescola.com.br, in the Apple Store, and on Google Play, with no time restrictions.

**Figure 1:**
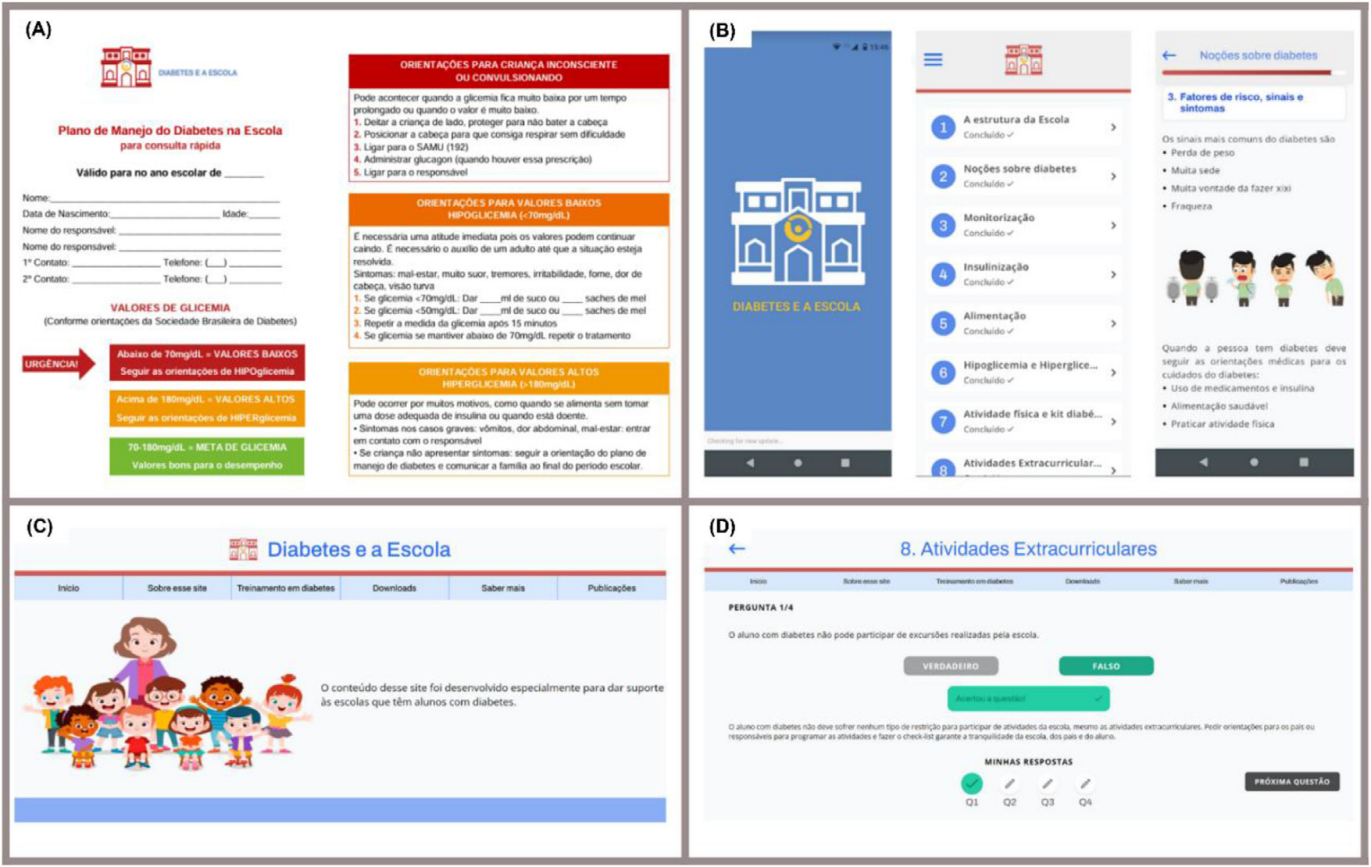
Educational package on diabetes care in schools. (A) Printed diabetes management plan. (B) Mobile application containing modules on diabetes training. (C) Website with training resources, downloads, and additional information. (D) An interactive platform with quizzes to reinforce learning.

The DMP was specifically developed for the project, based on the American Diabetes Association (ADA) and the International Society for Pediatric and Adolescent Diabetes (ISPAD), and adapted to the Brazilian school routine and the existing legislation [10,11]. The website also offered free downloads of the printable DMP, a "Diabetes Check-List," an "Extracurricular Activities Check-List," and a Quick Guide for Emergencies. This educational package remains available online after the conclusion of the research project (Figure. 1).

### Data collection and statistical analysis

Currently, there are no validated questionnaires for assessing diabetes care in schools, so the questionnaires used in this research were developed based on the existing literature, adapted to the Brazilian school routine, and applied in Portuguese [12,13].

Data collected from families of CwD consisted of sociodemographic variables and domains related to diabetes care at school, including treatment practices, self-care, hypoglycemia management, food management, and communication with school personnel. The initial questionnaire comprised 39 closed-ended items with dichotomous or multiple-choice responses, and one open-ended item. The follow-up questionnaire comprised 12 multiple-choice items and one open-ended item.

School professionals were contacted by participating families and invited to take part in the training sessions. Those who participated in the “on-site training” completed a 13-item questionnaire assessing their roles within the school and their experience providing assistance to students with diabetes, particularly in situations of hypoglycemia and hyperglycemia.

All questionnaires are available as Supplementary Material.

All data were recorded on the REDCap platform and analyzed using SPSS version 13.0 (SPSS Inc., Chicago, IL, USA). In the descriptive analysis, categorical variables were presented as frequencies and percentages, whereas continuous variables were described by measures of central tendency (mean, median) and dispersion (standard deviation). Exploratory univariate analyses were conducted to assess associations between selected variables of interest. Associations between categorical variables were evaluated using chi-square tests, with a significance level of 0.05.

### Ethical approval

Ethical approval was obtained from the Human Research Ethics Committee of Santa Casa de São Paulo (approval number CAAE39288120.6.0000.5479).

## Results

A total of 154 CwD families were invited to enroll in research and received an educational package. Of those, 107 (69.5 %) also agreed to complete the forms assessing their experience with diabetes care during school hours.

The average age was 10.7 years (± 3.89), the diabetes diagnosis at the age of 5.4 years (± 3.27), and 62.6 % of children were diagnosed before age 6. The majority live in the São Paulo metropolitan area (94.3 %; 27 cities), attended public school (89.7 %), and were in elementary school years (67.3 %). Mean HbA1c was 8.8 % (± 2.1; = 75/107).

Participantscharacteristics are shown in Table 1.

**Table 1:** Sociodemographic, clinical, and school setting characteristics of CwD included in the study.

Variable	n	%
Gender		
Female	46	43.0
Male	61	57.0
Age group		
≤ 6 years	20	18.7
7–10 years	31	29.0
11–14 years	32	29.9
≥ 15 years	24	22.4
Age at diagnosis		
≤ 6 years	67	62.6
7–10 years	30	28.0
11–14 years	10	9.3
School type		
Public	96	89.7
Private	11	10.3
School level		
Preschool/Kindergarten	13	12.1
Elementary (*Fundamental*)	72	67.3
High school	22	20.6
Respondent		
Mother	94	87.9
Father	8	7.5
Other	4	4.6
Place of residence		
São Paulo (capital)	58	54.2
São Paulo metropolitan area	43	40.1
Other cities	6	5.6
Type of treatment		
Basal/bolus (MDI)^a^	96	89.7
Insulin pump	11	10.3
Monitoring		
Blood glucose testing	95	88.8
Sensor (CGM^b^ or pump)	12	11.2

**Notes:** Percentages are based on valid responses.

a MDI-multiple daily injections. ^b^ CGM, continuous glucose monitoring.

### Diabetes management during school hours

The majority of CwD monitor their glucose levels (88.8%; 95) and inject insulin (89.7 %; 96) in school. Of the total, 12.1% (13/107) did not monitor their blood glucose at school due to factors such as lack of supervision, school restrictions, or personal choice, and 11.2% (12/107) reported that they were not allowed to bring diabetes care materials to school.

Regarding supervision, 43% (46) of all students administered insulin without supervision. Overall, 25.2% (*n*= 27) reported that a family member attends school to administer insulin, which was significantly more common in children under 6 years old (55 %, *n* = 11), followed by children aged 7 to 10 years old (41.9 %, *n* = 13).

When asked about the possibility of a school professional, who was not a health professional, assisting in diabetes care, 34.6 % would accept and offer to teach how to do it. In comparison, 27.1 % would accept only if training was provided for this purpose. In the group of children under 6 years of age, 45 % of parents/carers would agree to train school personnel, and 20 % would require a healthcare professional for the training (0.05).

### About the school's diabetes care structure

Regarding school experiences related to diabetes management, 10.3 % (*n* 11) of children had changed schools at least once due to diabetes care, and 7.5 % (8) reported that the school had never officially communicated with the family about the diagnosis or management of diabetes.

Most CwD (68.2 %; = 73) did not have a school staff member responsible for diabetes care. When broken down by school type, 72.7 % (*n* = 8/11) of CwD in private schools had at least one designated person, compared to 27.1 % in public schools (*n* = 26/96; *p* < 0.05).

The majority (65.4 %; *n* = 70) did not have a written emergency plan to guide the school in hypoglycemia care, and almost a quarter (24.3 %; *n* = 26) have experienced severe hypoglycemia at school. However, when asked about guidance from the health team on diabetes management during school hours, 50.5 % reported that they had provided instructions to the school.

Regarding contact with the school due to diabetes care, 40.2 % (*n* = 43) of families reported daily contact, and among the group of children under the age of 10, this percentage was as high as 70.5 % (*n* = 36/51; *p* < 0.05).

When asked about the school's knowledge of diabetes, 73.8 % (*n* = 79) considered it to be little or no knowledge. Regarding satisfaction with the school's diabetes management, only 22.4 % (*n* = 24) reported being completely satisfied, while 31.8 % (*n* = 34) reported being not satisfied, and 45.8 % (*n* = 49) reported being satisfied but believed they could still improve.

**Figure 2:**
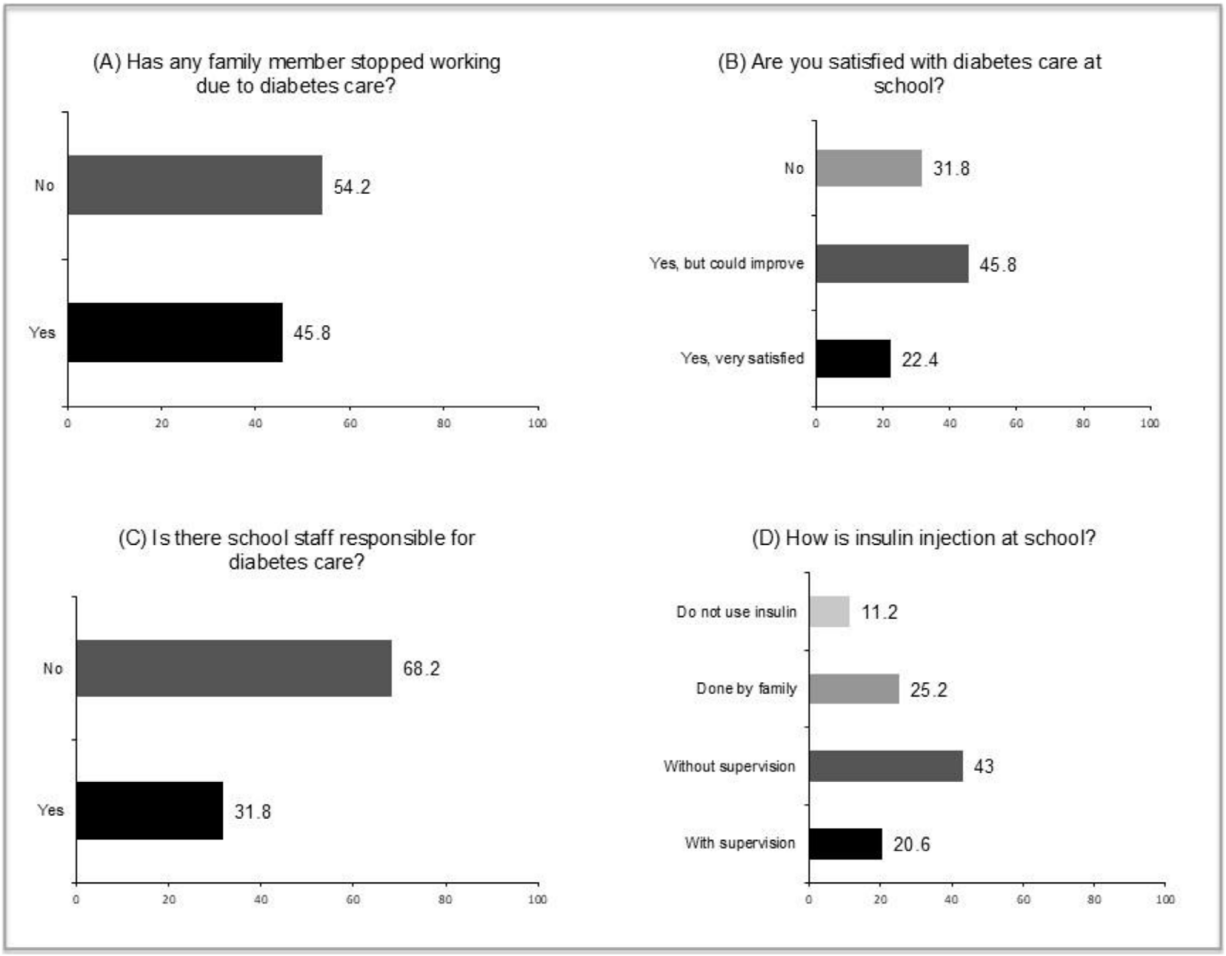
Family perceptions regarding diabetes care in schools.

**Note:** All values are presented as percentages.

A summary of families' perceptions of diabetes care in schools is shown in Figure 2.

### Evaluation after 4 months of intervention

The authors were able to reach 39 (36.4 %) participants, who completed the questionnaire four months after the intervention.

Regarding the educational package, most respondents (74.4 %, *n* = 29/39) reported discussing the content and believed the school valued receiving the material. When asked about their experience after delivering the educational package, 41 % (*n* = 16/39) felt that diabetes care at school improved, especially the relationship between the family and the school (25.6 %, *n* = 10/39) and the knowledge to correct hypoglycemia (20.5 %, *n* = 8/39).

As for negative experiences, one school did not accept the material at all; five participants reported that the school was initially resistant but accepted it; and one family felt that the relationship with the school had deteriorated afterwards.

### School participation

A total of 154 families of CwD received the educational package addressed to the schools. Three schools identified themselves after receiving the materials: one via the online form, another through the form plus onsite training, and a third during scheduled training.

There were a total of 388 website accesses and 286 app downloads (Apple Store: *n* = 139, Google Play: *n* = 147). It was not possible to collect data regarding the use of digital educational material.

In total, nine school professionals from two schools participated in the onsite training - one was a healthcare worker and therefore excluded from analysis. Among the eight remaining staff members, all directly involved in the daily care of students with diabetes, 62.5 % (*n* = 5) reported not knowing how to recognize symptoms of hypoglycemia or hyperglycemia, and 50 % (*n* = 4) did not know how to act if a child required assistance. Despite these gaps, 62.5 % (*n* = 5) had previously supervised capillary glucose testing, and 75 % (*n* = 6) felt comfortable doing so. Similarly, 37.5 % (*nn* = 3) had supervised insulin administration, and 87.5 % (*n* = 7) stated they would feel comfortable supervising insulin injections during school hours.

## Discussion

To the best of our knowledge, this was the first time an APP was developed for diabetes care training for school personnel in Brazil [14]. Previous studies have examined online training using either a website or a dedicated online training system. It has been documented that online training can be effective in diabetes care, even for individuals without prior healthcare training [15,16]. The primary goal of developing a new tool was to ensure its availability at all times. Cell phone use is widespread in Brazil, and a simple-to-use APP that consumes minimal memory and can be accessed offline would be an effective way to reduce barriers to access for diabetes care training.

Diabetes care faces disparities across the country, as well as within large cities like São Paulo and its metropolitan area [17]. In this cohort, half of the responses come from different cities with varying municipal human development index (IDH-M), making the sample diverse and providing opportunities to interpret data from a group of patients treated within the same healthcare system unit.

Over half of respondents reported no school staff member responsible for diabetes care, consistent with prior Brazilian studies [6,18,19]. Designated personnel were more common in private than public schools, suggesting socioeconomic differences in access to resources.

The percentage of families that stopped working is higher among CwD younger than 6 years (55.2 % vs 45.8 % overall), aligned with other studies [5,20]. Families of young CwD also need to go to school more frequently to inject insulin (55 % in those younger than 6 years vs 25.2 % of all). It's relevant to note that the majority of this population was diagnosed before age 6 years, and it´s well established that achieving and maintaining glycemic targets during the first 5 years after diagnosis is crucial for preventing long-term health problems [10,11].

School personnel generally respond positively when asked about diabetes training [6,21]. In this study, educational packages were provided across various platforms and most families reported that schools were glad to receive the material. Measuring impact is challenging, as improvements might be subjective and subtle. Previous training programs documented that completion is more likely among staff already responsible for a CwD, with on-site sessions preferred for experience sharing, although online formats remain more convenient [14,16].

As in international publications, a high percentage of CwD manage their diabetes without supervision [[22], [23], [24]]. Diabetes self-management depends more on developmental and psychosocial skills than on age or time since diagnosis. Even capable students should not be solely responsible; adult supervision is essential, especially in emergencies [3,4].

In Brazil, diabetes-related procedures such as blood glucose monitoring or insulin administration by non-healthcare personnel during school hours are neither regulated nor generally permitted. The lack of official requirements for supporting students with diabetes may explain the observed low adherence to training. Most schools lack first aid stations or nurses, relying instead on student self-management [6,22]. In some cases, staff voluntarily assist children during school hours, even without formal obligation [18].

It has been reported in national and international publications that families have needed to change their children’s schools due to challenges related to diabetes care [6,20]. In Brazil, policies supporting students with chronic conditions are fragmented, relying on broad inclusion laws, federal programs, and regional regulations, with significant variability in implementation [[25], [26], [27]]. All regulations state that schools must be prepared to support the well-being of children during school hours; however, it is unclear how this should be implemented regarding diabetes care. In São Paulo state, public education is managed by both municipal and state governments and is divided into regional administrative units. While schools follow the same legal and curricular frameworks, differences in local management, funding, and neighborhood socioeconomic profiles lead to variations in infrastructure, staffing, and program availability across regions. A 2019 state law requires schools to train staff to support students with special needs, including those with T1D. However, it does not specify who is responsible for administering insulin or monitoring blood sugar levels [27].

Over 60 % of families would accept diabetes care from trained non-medical staff, instructed by family or healthcare providers. School personnel are usually exposed to diabetes management only when a student is diagnosed, with families guiding them [6]. In Brazil, the KIDS pilot showed staff attitudes improved after learning about diabetes. Both trained schools supported students and aimed to improve knowledge. Internationally, the lack of school health providers, especially nurses or diabetes educators, remains a challenge [13].

ISPAD and ADA recommend that healthcare professionals collaborate with families to develop individualized DMPs, enabling schools to meet each child’s needs [3,4]. However, written DMPs are often absent; in this study, families reported delivering instructions from healthcare to the school but not as a formal DMP [23,24]. Even when families discuss diabetes care with the school, caregivers and students feel that school personnel still cannot properly manage a diabetes emergency [22,28]. Some schools declined to receive DMPs, and some caregivers reported a deteriorated relationship with the school after intervention. This may reflect increased pressure perceived by schools when official documents clearly outline responsibilities.

Different countries showed that law changes have an impact on diabetes care during school hours [5]. Sweden, as an example, documented that the percentage of caregivers reporting that their child had an action plan to treat hypoglycemia increased from 55 % to 65 %, following the introduction of a national uniform action plan for all schools [29].

### Limitations

The study was conducted at a university hospital, which may limit generalizability beyond the São Paulo metropolitan area. However, Santa Casa de Misericórdia de São Paulo primarily serves patients from the Brazilian public health system, who are predominantly from lower socioeconomic backgrounds. In addition, nearly half of the participants resided in 27 other cities, and the majority attended public schools, suggesting that the sample is representative of this population.

The educational material was created using tools that have already been validated in printed form, but validation has not been performed for the website or APP formats. However, this educational package represents a meaningful step forward in providing schools with access to trustworthy information tailored to their specific needs. Conducting studies to validate this mode of educational training would be highly beneficial.

The response rate to the follow-up family survey limited the ability to directly compare pre- and post-intervention outcomes at the individual level. In addition, the study was not designed to evaluate the effectiveness of the intervention but rather to assess the acceptability, applicability, and participants' experiences with the educational package provided.

School engagement may be interpreted as a limitation of the study; however, it also represents a relevant finding in itself, reflecting the challenges of engaging schools in diabetes related training initiatives. In addition, difficulties in tracking access to digital training platforms should have been anticipated and represent an inherent limitation of the intervention design.

To our knowledge, this is the first study to assess diabetes care in the São Paulo metropolitan area, develop an educational training platform in APP format in Brazil, and document the outcomes of providing diabetes training to school personnel. The findings highlight an urgent need to strengthen diabetes care in schools, as families often compensate for the lack of institutional support by interrupting their work to provide care during school hours, with a greater impact on those with younger children and in public schools. The educational package developed in this study proved valuable in making reliable information more accessible and showed potential to improve school-based diabetes care. Efforts must be made in collaboration with the public educational system to facilitate the engagement of school personnel in managing diabetes during school hours.

## Funding sources

This work was supported by the São Paulo Research Foundation (FAPESP, grant number 2021/07119-0) and by Santa Casa de São Paulo Research Support Fund (FAP, grant number 009/21-23). These grants supported the design of the digital platforms (website and mobile application) and the dissemination of the project through printed and online media

## Data availability

Data are not publicly available due to ethical and confidentiality restrictions, but may be obtained from the corresponding author upon reasonable request.

## CRediT authorship contribution statement

**Laura Cudizio:** Conceptualization, Methodology, Data curation, Writing – original draft, Project administration. **Isadora R. Brambilla:** Data curation, Writing – review & editing. **Kayleigh G. Marques de Araújo:** Data curation, Writing – review & editing. **Raquel G. Lot:** Data curation, Writing – review & editing. **Tayana O. Martins:** Data curation, Writing – review & editing. **Raquel R. Biasi:** Data curation, Writing – review & editing. **Giuliane Galeote:** Data curation, Writing – review & editing. **Aline D. Costa-Riquetto:** Writing – review & editing. **João Eduardo N. Salles:** Conceptualization, Funding acquisition, Supervision, Writing – review & editing. **Luis Eduardo Calliari:** Conceptualization, Methodology, Data curation, Writing – original draft, Funding acquisition, Supervision.

## Conflicts of interest

The authors declare no conflicts of interest.

## Appendix Supplementary materials

Image, application 1
