# Supplementary material for: Families’ perspectives and the development of an educational program for diabetes in schools
Source: J Pediatr (Rio J). 2026 Feb 28;102(2):101512. doi: 10.1016/j.jped.2026.101512 (PMC12966706; doi:10.1016/j.jped.2026.101512)
Supplement: Supplementary file 1 [file mmc1.docx]

**JPED-D-25-00406**

**Supplementary material**

**1. Questionnaire – Families of children with diabetes**

1. Municipality:

2. Postal Code:

3. What is your relationship with the child with diabetes?
☐ Mother
☐ Father
☐ Grandparent
☐ Sibling
☐ Other

4. Child’s current age (in years):

5. Age at diabetes diagnosis:

*(e.g., 10 months, 5 years)*

6. How would you best describe your child?
☐ Female
☐ Male
☐ Other
☐ Prefer not to answer

7. School Level
☐ Daycare (< 4 years)
☐ Preschool (> 4 years, before 1st grade)
☐ Elementary (1st–9th grade)
☐ High School
☐ Technical School

8. Type of School
☐ Public
☐ Private

9. Are there other children with Type 1 Diabetes at the same school?
☐ Yes
☐ Not currently, but there have been before
☐ No
☐ I don’t know

10. Has the child ever changed schools due to diabetes care?
☐ Yes, once
☐ Yes, more than once
☐ No

11. Has any caregiver in your family stopped working due to diabetes care?
☐ Yes
☐ No

12. Do you believe your child experiences discomfort at school due to diabetes (e.g., bullying, isolation, exclusion, stigma)?
☐ Yes
☐ No

13. How does the child check blood sugar at school?
☐ Finger prick
☐ Libre sensor
☐ Glucose sensor attached to insulin pump
☐ Doesn’t check at school due to lack of supervision
☐ Not allowed to check at school
☐ Doesn’t want to check at school

14. Can the child check glucose levels independently?
☐ Yes, without supervision
☐ Yes, with supervision
☐ No

15. How does the child administer insulin?
☐ Syringe
☐ Pen
☐ Syringe/Pen (depends)
☐ Continuous Infusion Pump

16. Can the child administer insulin independently?
☐ Yes, without supervision
☐ Yes, with supervision
☐ No

17. What basal insulin does the child use?
☐ NPH
☐ Glargine (Lantus, Basaglar, Glargilin)
☐ Detemir (Levemir)
☐ Degludec (Tresiba)
☐ Infusion Pump
☐ I don’t know

18. What bolus insulin does the child use?
☐ Regular
☐ Ultra-rapid (Lispro, Humalog, Aspart, Novorapid, Glulisine, Apidra)
☐ FIASP
☐ I don’t know

19. How is the insulin dose for meals prescribed?
☐ Fixed dose for each meal (at home and school)
☐ Chart based on glucose level (at home and school)
☐ Chart based on glucose level, but fixed dose at school
☐ Carb counting at home, chart based on glucose level at school
☐ Carb counting always (at home and school)

20. How is the bolus determined at school?
☐ Child determines and administers insulin dose independently
☐ Child determines the dosage with supervision
☐ School staff contacts me every time to confirm the dose
☐ School staff follows the prescription and choose the dose
☐ I inform staff of the dose based on what the child will eat
☐ I go to school to administer insulin
☐ My child does not take insulin at school

21. Has insulin treatment been modified due to lack of support at school?
☐ Yes
☐ No

22. How often do you reduce insulin dose due to fear of hypoglycemia at school?
☐ Every day
☐ Once a week
☐ Once a month
☐ Never

23. Does your child participate in extracurricular activities (e.g., school trips)?
☐ Yes, with my supervision
☐ Yes
☐ No
☐ School does not allow participation due to diabetes
☐ School does not offer such activities

24. Has your child ever had a severe hypoglycemia episode at school?
☐ Yes
☐ No

25. Does the school have a written emergency plan for hypoglycemia?
☐ Yes
☐ No
☐ I don’t know

26. Is the school’s food appropriate for your child?
☐ Yes
☐ No
☐ My child does not eat school food

27. Does your child have access to special foods when needed (e.g., gluten-free)?
☐ Yes
☐ No
☐ My child does not eat school food

28. Is there a staff member responsible for diabetes care at school?
☐ Yes, one person
☐ Yes, more than one person
☐ No

29. How often do you keep your child home from school due to diabetes?
☐ Once a week
☐ Once a month
☐ Once a year
☐ Never

30. Are you satisfied with the diabetes care your child receives at school?
☐ Very satisfied
☐ Satisfied, but it could improve
☐ Not satisfied

31. Have you ever felt discriminated against by school staff due to diabetes?
☐ Yes
☐ No

32. Would you accept help from a non-health professional school staff member?
☐ Yes, if trained
☐ Yes, I would teach them
☐ Not necessary, my child is independent
☐ No
☐ The school already has someone responsible

33. Is there an extra staff member at school due to diabetes (e.g., nurse)?
☐ Yes, hired specifically for diabetes care
☐ Yes, already present at school
☐ No

34. Is there a suitable place at school for checking glucose/applying insulin?
☐ Yes
☐ No
☐ I don’t know

35. Is there a suitable place to store insulin and supplies at school?
☐ Reserved space
☐ In child’s backpack/locker
☐ School does not allow supplies
☐ I don’t know

36. What is your opinion of school staff’s knowledge about diabetes?
☐ No knowledge
☐ Little knowledge
☐ Average knowledge
☐ Good knowledge
☐ I can’t assess

37. Does your healthcare team provide a Diabetes Care Plan for the school?
☐ Yes
☐ No
☐ I don’t know

38. How often do you communicate with the school about diabetes care?
☐ Daily
☐ Weekly
☐ Monthly
☐ Only when child complains
☐ Only when teacher contacts me
☐ Never, but I wish I could
☐ Never, and I’m okay with that
☐ School has never officially contacted me about diabetes

39. How do you communicate with the school? (Select all that apply)
☐ Phone (calls or texts)
☐ Email
☐ Parent-teacher meetings
☐ Talking during drop-off/pick-up
☐ Notebook/diary/app with notes

40. Would you like to share any experiences with us?
*(All responses are anonymous and no one will contact you.)*

**2. Questionnaire to be completed by the school staff**

| 1 | Date |
| --- | --- |
| 2 | School |
| 3 | Municipality/State |
| 4 | The school you work in most of the time is:   1. Municipal 2. State 3. Federal 4. Particular |
| 5 | What is your role in the school?   1. Direction 2. Coordination 3. Teaching staff 4. Physical education 5. Monitoring 6. Nutritionist 7. Pedagogy 8. Psychology 9. Healthcare professional 10. Other__________________________________ |
| 6 | Have you ever had contact with Type 1 Diabetes Mellitus?   1. Yes, I have/have had students with diabetes 2. Yes, in my family or with close friends 3. Yes, by social media or television 4. No, this is the first time |
| 7 | In your opinion, would training be useful to improve your relationship with your student and family?   1. Yes 2. No |
| 8 | If you have a student with diabetes currently, do you know if the child has written guidance given to the school for diabetes care?   1. Yes, there is a written guideline 2. No, there is no written guidance 3. I have no knowledge 4. I don't have any students diagnosed with diabetes currently |
| 9 | How do you feel about supporting the care of children with diabetes while you are in school? (if you don't have this experience, imagine how you would feel)   1. It's challenging, but it's not a problem 2. I feel worried and tense 3. I feel uncomfortable and intimidated |
| 10 | How do you consider your knowledge of diabetes?   1. None 2. Little 3. Regular 4. Good 5. Very good |
| 11 | How do you consider your fear of high blood glucose in a student with diabetes?   1. I have no experience with diabetes in school 2. I'm not afraid 3. I'm very afraid 4. I don't know what it means to have high blood glucose |
| 12 | How do you consider your fear of low blood glucose in a student with diabetes?   1. I have no experience with diabetes in school 2. I'm not afraid 3. I'm very afraid 4. I don't know what it means to have low blood glucose levels |
| 13 | How would you feel if you were asked to help care for a student with diabetes while he or she is in school?   - 1. I would not accept to take on this responsibility   2. He would agree to help as long as he received training   3. This responsibility should be that of a designated health care professional   4. I have no opinion about it |

**3. Families follow-up survey**

1. School Year*
   - Nursery (<4 years old)
   - Preschool (older than 4, but before starting elementary school)
   - Elementary (Grades 1–9)
   - High School
   - Technical School
2. Type of School*
   - Public
   - Private
3. Did you deliver the Diabetes Management Plan (DMP) to the school?*
   - Yes
   - No
   - I don’t know
4. Do you know what the school did with the DMP?*
   - Stored it
   - Made a copy and returned it
   - Did not accept it
   - I don’t know
5. Did you discuss the DMP with the school?*
   - Yes
   - No
6. Do you think the school appreciated receiving the material?*
   - Yes
   - No
   - I don’t know
7. Do you think anything has IMPROVED in the child’s diabetes care?*
   - Yes
   - No
   - I thought diabetes care was already good
8. What do you think IMPROVED in diabetes care at school?
   *(Check all that apply)*
   - Blood glucose monitoring (appropriate location, better support)
   - Insulin administration (appropriate location, better support)
   - Hypoglycemia correction
   - Relationship with the school
   - Nutrition
   - Nothing improved
   - Everything was already good
9. Do you think anything has WORSENED in the child’s diabetes care?*
   - Yes
   - No
10. What do you think WORSENED?
    *(Check all that apply)*
    - Blood glucose monitoring (appropriate location, better support)
    - Insulin administration (appropriate location, better support)
    - Hypoglycemia correction
    - Relationship with the school
    - Nutrition
    - Nothing worsened
    - It was always bad
11. Are you satisfied with the diabetes care at school?*
    - Yes, very satisfied
    - Yes, but I think it could improve
    - No
12. What do you think about the school staff’s knowledge of diabetes?*
    - They have no knowledge
    - Little knowledge
    - Average knowledge
    - Good knowledge
13. Would you like to share any experience with us?
    *(All responses are anonymous and no one will contact you)*

.
